# Mobile Poultry Processing Unit as a Resource for Small Poultry Farms: Planning and Economic Efficiency, Animal Welfare, Meat Quality and Sanitary Implications

**DOI:** 10.3390/ani8120229

**Published:** 2018-11-30

**Authors:** Alice Cartoni Mancinelli, Alessandro Dal Bosco, Simona Mattioli, David Ranucci, Cesare Castellini

**Affiliations:** 1 Dipartimento di Scienze Agrarie, Alimentari ed Ambientali, Università di Perugia, Borgo 20 Giugno, 74, 06123 Perugia, Italy; alessandro.dalbosco@unipg.it (A.D.B.); simona.mattioli@hotmail.it (S.M.); cesare.castellini@unipg.it (C.C.); 2 Dipartimento di Medicina Veterinaria, Via San Costanzo, 4, 06126 Perugia, Italy; david.ranucci@unipg.it

**Keywords:** Mobile Poultry Processing Unit, pastured poultry production, animal welfare, meat quality, economic efficiency

## Abstract

**Simple Summary:**

Poultry meat production is now based on fast-growing strains, with consequences for animal health and welfare. There is also an increasing demand for products from extensive rearing systems; there are, however, several criticisms including the difficulty of slaughtering chickens from a logistic, legislative and economic point of view. A possible solution could be represented by the use a Mobile Poultry Processing Unit (MPPU), which directly reaches the poultry farms. The aim of this review is to analyse the requisites and economic efficiency of a MPPU prototype in Italy; further, the related animal welfare aspects and the qualitative and sanitary implications are discussed.

**Abstract:**

Nowadays there is an increasing demand for poultry products from alternative rearing systems. These systems, commonly named pastured poultry production (PPP), are more expensive than intensive rearing system but sustain biodiversity, local economies and farm multi-functionality besides providing meat to which consumers attribute high ethical value and quality. PPP generally uses large outdoor runs, small number of animals and requires chickens adapted to natural environment. One of the most relevant obstacles to further development of PPP systems is related to the slaughtering of animals economically and at the same time complying with the sanitary regulations to maintain food safety standards. A possible solution could be represented by a Mobile Poultry Processing Unit (MPPU), which directly reaches the poultry farms. MPPU can consider a good compromise for the niche production providing an opportunity to small farmers to exploit the full potential of their production system. The aim of this review is to analyse the essential requisites and MPPU economic viability in an Italian system. Qualitative, societal aspects are discussed together with bird welfare and hygiene implications. The case study indicates the viability of MPPUs but notes that up scaling to medium sized operations would not be permissible under current EU regulations.

## 1. Introduction 

Poultry production has a lower environmental impact when compared to other livestock production chains [1], mainly due to its high efficiency in converting feed into meat. The main reason for this high efficiency ratio is related to the strong genetic selection carried out to increase productive performance. Modern broilers reach their slaughter weight earlier than ever before, with a high yield of breast and other meat [2]. Unfortunately, these fast-growing strains can show welfare and health problems, skeletal imbalance [3], metabolic disorders, myopathy and other muscle disorders [4], which affect the appearance of the meat, nutritional traits and consumers acceptance [5]. 

A side-effect of this process is the reduction of genetic variability [6] and the vulnerability of these chickens to environmental stress [7]. Nevertheless, poultry meat production of western countries is based on these chicken strains and it is now accompanied by a growing concern for the health and welfare of these animals [8]. As a result, there is an increasing demand for poultry meat produced in extensive rearing systems [9]. These production systems, commonly named pastured poultry production (PPP), are more expensive than intensive systems but can help sustain biodiversity, local economies and farm multi-functionality, in addition to providing meat to which consumers attribute a high ethical value, quality and taste [10]. Indeed, PPP generally uses large outdoor runs (at least 4 m^2^/chicken), small number of animals and requires chickens adapted to a variable environment, without the strict control of temperature, humidity and ventilation of in the intensive systems [11].

It is widely known that the access to free-range areas greatly improves the welfare of poultry and that the presence of shrubs and trees in the pastures further increases the use of runs [12]. For these reasons, the PPP is often found in an agro-forestry production system (such as fruit orchards or olive orchards). This combination improves the environmental sustainability of production because two or more different productions (meat, fruit and crops) can be simultaneously obtained from the same land, providing advantages both for the chickens and for the orchard. 

Chickens and orchards mutually benefit each other; chickens improve soil quality by adding organic matter and control both insect pests and weeds, while trees protect the chickens from adverse weather conditions as well as raptors and provide additional revenue for the farm [13].

Even though the pasture offers only a modest supply of energy and proteins—the pasture in PPP represent only 10–15% of the total feed intake—it provides many bioactive compounds, such as xanthophylls, antioxidants and vitamins [14,15]. Accordingly, the meat from PPP may have some nutritional benefits compared to standard broiler meat [16]. Several authors [17,18] reported that bioactive compounds are transferred from the pasture to chicken meat, as shown by a higher meat content of antioxidants and n-3 polyunsaturated fatty acids. Additionally, access to pastures may contribute to meat flavour, with some forage and herbs resulting in distinctive flavours [19,20].

As previously reported, PPP requires chickens that are adapted to the natural environment (high kinetic activity and foraging behaviour), with a well-developed immune system and adequate body conformation and skeletal development [11,21,22,23]. Previous research has shown that slow-growing chickens are more adaptable to outdoor runs due to suitable thermo-tolerance, foraging aptitude, immune response and antioxidant capacity compared to fast-growing strains [24,25,26]. In contrast, fast-growing broilers selected for intensive production systems are fit for living in controlled conditions (controlled environment, veterinary care and diets high in protein and energy) and do not adapt to PPP. Dal Bosco et al. [10] compared behaviour in the chickens and showed that slow-growing chickens covered an average daily distance of 1130 m, while fast-growing ones walked only 220 m. 

Contrary to the popular belief, there are several disadvantages with respect to PPP that preclude further development and reduce the production capacity of this system. The main disadvantages are reported below:

**Cost of production**—The production cost of this meat is much higher than with a conventional system, mainly due to the lower growth performance and the breast meat yield of slow-growing genotypes.

**Risk of predation**—PPP systems are attractive to predators (foxes, birds and other wild animals). Permanent fencing is expensive and is not always effective at excluding predators from the pasture. 

**Rules formulated for large-scale poultry farms**—Sanitary rules and technical standards of poultry production are often based on large-scale farms. Small farmers cannot afford to invest in requirements and protocols unsuited to their system of production [27].

Consequently, up and down-stream infrastructures (e.g., hatchery with genetic strains adapted to PPP or slaughter houses), services (know-how or vaccines for small number of birds) are lacking. In particular, one of the major bottlenecks is the slaughterhouse. During the last 20 years in western countries, there has been a huge decrease in the number of poultry slaughterhouses coupled with an increase in size of the existing ones. Moreover, nowadays slaughterhouses generally belong to food companies and are not available to other farms. 

The lack of slaughterhouses strongly discourages the use of PPP and many farmers limit their production to less than 500 or 1000 birds per year. This quantity, according to European (853/2004) and USA (62) regulations, is considered to be primary production which can be sold directly to consumers without controlled slaughter. However, the restrictions on sales and the lack of small-scale poultry slaughterhouses prevent the creation of specialised poultry farms. Small-scale farmers who wish to sell poultry products locally must have them slaughtered and processed in inspected facilities that are usually far from the farms.

A possible solution which increases the use of PPP systems could be a Mobile Poultry Processing Unit (MPPU) mounted on a small truck or van which goes directly to the poultry farms. A MPPU is excluded from continuous inspections by the Food Safety Authorities but it is still required to meet all sanitation and requirements. These MPPUs are designed to eliminate regulatory impasses and increases marketability and profitability for small-farmers. The MPPU reaches the farm directly on the day of slaughtering and then another advantage is the absence of transport of live animals to the slaughterhouse. This positively influences animal welfare and meat quality (see Section 5 and Section 6).

The main characteristics of MPPU are described later in the Section 2 and Section 6 and pros and cons are summarized in Table 1.

Beside the economic aspects, there are also social aspects connected with the use of PPP and MPPUs. In a sound productive chain, the system of production should be followed by a coherent transfer of goods and services (from farm to consumer). Each productive system should develop a proper system. For example, intensive poultry production has developed not only a proper production system but also a typical structure for exchange. Accordingly, standards arising from industrialised agriculture may be at odds with the principles of small-scale poultry systems [28].

Rural sociology suggests a general framework in which exist a correspondence between production systems and the structure for exchange. PPP, as other local production system, requires the creation of specific short and decentralised circuits that link the production with the consumption of food. This pattern is completely different from highly centralised paths constituted by large food processing and trading companies which operate on a large scale [29]. 

Our opinion is that the exchange structures promoted by industrialised processing may not be able to handle all the benefits of PPP and they could negatively influence its internal equilibrium [29]. Conversely, PPP is not able to exploit the benefits of the large-scale supply systems. Accordingly, PPP requires specific approaches to solve marketing issues; these approaches cannot be derived from the experience of other productive systems.

According to this view, a MPPU could be considered not only a solution to the slaughtering problem but also a resource to enhance the emergence of food circuits (e.g., farmers’ markets and niche markets), which cannot feature within the global food chains [30,31,32]. Indeed, a MPPU can contribute to managing this emerging circuit, allowing the creation of a production chain which exploits the ethical value adapted to its dimension value, prize. 

Moreover, through use of a MPPU, the cost of each farm owning a slaughterhouse (€50–80,000) is avoided. European legislation recognises and authorises on-farm slaughter but the slaughterhouse must be used only for animals raised on the farm. This causes high initial investment for each farmer. In the light of what is reported, the aim of this paper is to review the current MPPU technologies by examining:

- Essential requirements of a MPPU
- Operation and economic efficiency
- Animal welfare aspects
- Qualitative and sanitary implications.

In the USA, several types of MPPU have been available for about 10 years [33,34], whereas in Europe, mobile slaughtering facilities are rare and only a few data are available. Accordingly, the present review will focus mainly on EU regulations and the EU situation.

## 2. Essential Requirements: Planning and Layout 

During the last 10 years, National Authorities have pointed out rules to allow the activity of these MPPU. The basic principles of slaughtering in the EU include the need to fulfil several requisites, specified in the Regulation EC/853/2004 (Annex II, Chapter I, III and IV point 1) and by the Sanitary Authority which eventually authorises the use of the MPPUs on different farms. In this way, it is possible to share the MPPU among several farms, thus reducing the slaughtering cost of each chicken.

Furthermore, the structure needs to be properly designed to avoid cross-contamination and it should be placed in a specific area of the farm where drinking water and electric power are available (unless a drinking water tank and a generator are present to directly supply the MPPU), regular pest control is performed and waste (water and slaughter by-products) can be easily managed.

The MPPU must be built with materials and equipment that are easy to clean by Sanitation Standard Operating Procedures (SSOP) and there must be adequate site management and appropriate personnel hygiene and clothing. Personnel operating in the MPPU must be trained in slaughter procedures and a proper HACCP (Hazard Analysis and Critical Control Points) plan needs to be implemented in the MPPU.

With regard to the slaughter procedure, stunning, bleeding and plucking must be performed separately from evisceration. Stunning must be performed according to Regulation EC/1099/2009. Chilling and storage of carcasses and meat must take place immediately in the MPPU or on the farm. If chillers, generally static chillers, are available on the farm, they must be placed near the MPPU and a suitable system for protecting the carcasses during transport from the slaughter site to the chiller needs to be adopted to avoid exogenous contamination.

Animal by-products must be managed according to Regulation EC 1069/2009. Cleaning and disinfection of the MPPU must be performed at the end of each slaughtering session, eitherat the farm or in a specific staging area. Meat must be labelled with the date of slaughter, farm code and farm address. A proper traceability system has to be set up. 

Not all poultry farms have these facilities and an evaluation of the best site on the farm for slaughter, supply of water and management of wastewater, is needed. 

In the United States, the hygiene requirements and SSOP are almost the same as in the EU (Mobile Poultry Processing Unit Farm & Food Safety Management Guide, 2012). Although the local area is considered as the State and the maximum number of birds slaughtered is higher than 10,000.

The other restrictions imposed by law in the EU are:

- The MPPU can be used on poultry farms producing less than 10,000 birds per year (EU State-Regions) [35];
- The meat processed in a MPPU can only be sold in neighbouring areas (the province where a MPPU is located and adjacent areas, within 50 Km of the province border) and this is not valid for all States of the EU;
- The buyer should be a retailer, that is, selling directly to the consumer.

According to this set of rules, production from a MPPU is small, local and subjected to severe numerical and geographical restrictions, confirming this system as a close and small one.

At the short term, the geographical and numerical limitations imposed by EU regulations do not permit future development of MPPUs for medium- or large-scale production. Moreover, even if some technical (different stunning system, carcass decontamination and water bath chilling) or regulatory improvements (geographical limitation) of MPPUs were advisable, European legislation strictly limits further improvements. Indeed, the change of regulatory system, at least in EU, is very long and complex and needs multiple level of decision (European, National and National/Regional).

Nonetheless, other countries could take advantage of the European experience and improve MPPUs according to national legislation.

## 3. A Case Study of MPPU in Italy

**Figure 1:**
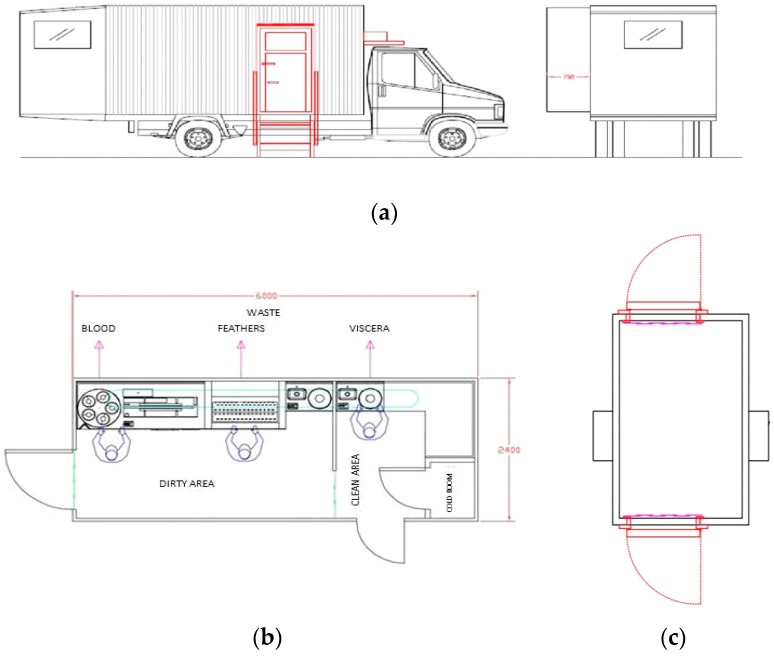
Schematic layout of the Mobile Poultry Processing Unit (50 chickens/hour): external (**a**) and internal (**b**) configuration and refrigerated trailer (**c**).

**Figure 2:**
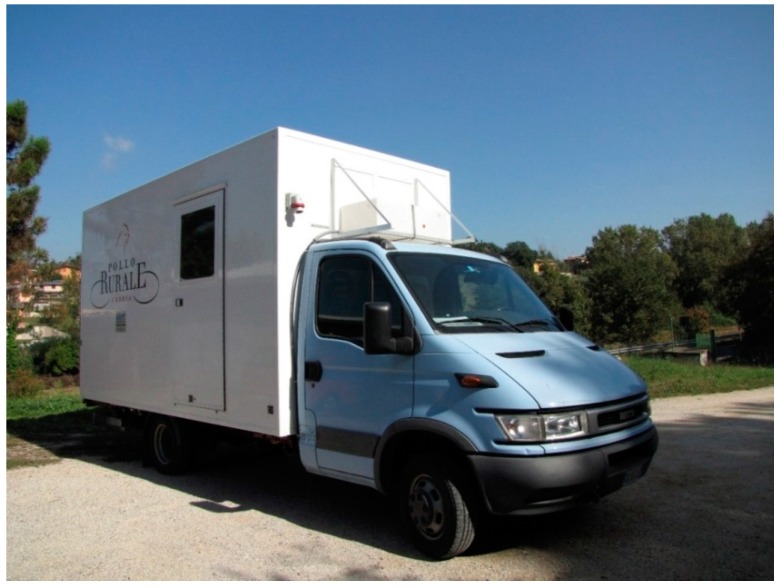
Photo image of a MPPU.

According to these requirements, a MPPU has been planned and built in central Italy (Figure 1 and Figure 2). This type of MPPU is the first in Europe and for this reason the study was carried out considering only whit this equipment.

The small dimension of this MPPU has made it possible to put the equipment in a small truck, which can be handled with a standard driving license. It is also possible to add a refrigerated trailer for the farmer to deliver the carcasses directly to the market after slaughtering.

The innovative point of this process consist in the absence of animals transport, in fact the MPPU reaches the farm the day of the slaughter improving the pre-slaughtering operations. The slaughter process begins by a withdrawing feed of 8–10 h and a sanitary control of animals. One hour before slaughter, all of the chickens are captured, caged and placed on the MPPU’s external platform (Figure 1a).

The truck is internally divided into two areas as required by the regulations:

- Dirty area;
- Clean area; so at least two operators are needed, one for each area.

The employee in the dirty area carries out all the operations from stunning to plucking; in particular he takes the animals one at a time from the cage, electrically stuns them and places them in the bleeding cone.

The subsequent phases involve the processing of four animals at a time that are placed in the scalder at a temperature of 58–60 °C for 30–60 s to loosen the feathers and in a drum plucker for 40–50 s.

At the end the carcasses are hung in hooks and transferred to the clean area where the second operator provides to the eviscerating. The final two steps are represented by the refrigeration and packaging. The working capacity of this MPPU is about of 50 chickens/hour.

## 4. Operational and Economic Efficiency

Since a MPPU requires substantial investment by the farmers, an evaluation of the economic feasibility of a MPPU compared to the current processing methods would allow farmers to plan their production method [34]. 

Different MPPUs—from basic to more automated and expensive ones—have been developed worldwide, mainly in the USA. Naturally, the cost of the equipment can vary substantially (from about €10,000 to 180,000). 

**Figure 3:**
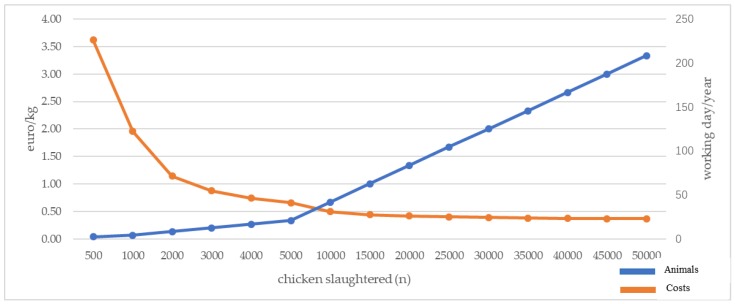
Slaughtering cost (€/kg) and number of working days according to the number of chickens slaughtered (our elaboration).

Starting from the case-study shown above, we calculated the cost of chicken processing (Figure 3). In our case-study, the MPPU was designed for a small truck (IVECO DAILY; l 5000 × w 2200 × h 2400 cm), whereas the dimensions of the refrigerated van were l 3240 × w 1550 × h 2105 cm.

The equipment for the slaughter of poultry was separated into two areas: “dirty area” and “clean area” (Figure 1). The “dirty area” comprises a platform for live animals, an electro-narcosis stunner, kill tank and cones, scalder, steriliser for knives, plucker and carcass guideway. The “clean area” comprises an evisceration table, a semi-automatic eviscerator, a steriliser for knives, a fridge, a table with a weighing scale and a generator.

As required by official controls on food safety, at least two people have to work in the MPPU, one in the clean area and the other in the dirty area. The number of birds/hour which can be processed is around 50.

The total cost of the equipment (truck + van) is roughly €150,000, which can be shared among different farms. In a case scenario of 10 farms, the cost would be reduced to about €14–15,000 per farm, compared with a cost of about €50–80,000 for a farm slaughterhouse. 

In addition, the MPPU cuts out the costs related to the transport of live animals from the farm to the slaughterhouse and the subsequent transport of the refrigerated carcasses back to the farm or stores. Typically, the transport of live animals and carcasses must be carried out by different vehicles, the first one complying with the regulations concerning animal welfare, the second one complying with the hygienic-sanitary requirements for meat which mainly involves refrigeration. 

It should also be taken into consideration that EU and National funds, which support the improvement of competition and the modernization of farm facilities, could partially cover (30–50% of the eligible cost) the purchase of a MPPU. 

It should be underlined that the avoidance of transportation permits to improvement in the welfare of chicken, see the next section. 

Naturally, the care of animals during catching and caging is also particularly important in a MPPU. To avoid excessive chicken shackling, the chickens should be caged just before slaughter. Caged animals are placed in the rear platform of the truck equipped with a cover, which serves as a rest area where the veterinarian can perform the pre-slaughter inspection of animals.

These costs are based on an actual Italian case study. According to Figure 3, when the number of chickens slaughtered is very low (<2000 per year) the cost is high (>€1/kg live weight processed), whereas the trend is for the cost to become consistently lower for numbers > 25,000. Accordingly, small farmers should efficiently plan the number of chickens to be slaughtered, with the possibility of collective use of the same MPPU. Other management options are also available (e.g., leasing and rent) and can make the use of MPPUs less expensive. 

Angioloni et al. [34] showed a similar trend (decreasing costs with increasing number of slaughtered chickens) and showed that ten farmers sharing the ownership of the MPPU can achieve a higher profit than using alternative off-farm inspected slaughter facilities. In the USA, current estimates show that the cost is variable but within a close range.

The cost of conventional slaughter includes:

- transport of live animals by authorised vehicle €1–1.50/chicken (depending on the distance of the slaughterhouse from the farm);
- slaughtering process 0.50 €/kg of live weight, therefore, an average of €1.50/chicken;
- transport of carcass by refrigerated vehicle, €1.50/carcass.

In summary, the cost of slaughter for each animal ranges from €3.50 to €4.50.

In conclusion, the economic analysis indicates the cost of slaughter using a MPPU is, on average, on the same scale as the cost of an on farm stationary processing system when the number of animals is higher than 10,000 year (the maximum admitted for this stationary plan) and lower than costs involved with a commercial slaughterhouse.

## 5. Animal Welfare Aspects

As previously reported, slaughterhouses are becoming bigger and the distance between farms and slaughterhouses is, in some cases, very large.

European law sets out several compulsory requirements on the transfer of poultry to slaughterhouses: density in the crates; allowing drinking and feeding if more than 12 h are needed to reach the abattoir; limits to faecal matter falling from animals in upper layers to the underlying crates; and temperature and ventilation in the trucks during transport. These rules mainly aimed at fast-growing broilers produced on intensive farms. However, very little data are available with respect to transport of birds from free-range systems [11]. It is expected that the more active animals used in PPP systems will respond to this transport stress differently to fast-growing chickens.

Poultry transport to the slaughterhouse is one of the critical factors affecting animal welfare, quality and meat hygiene. These different stressing situations can reduce bird welfare and increases the risk of body injuries (broken wings/legs and overall distress) and mortality [36]. Chickens are caught and placed in crates to reach the slaughterhouse and during the transport they have no feed and water, are exposed to environmental changes (i.e., movement, noise, vibration), subject to even extreme conditions of temperature and humidity, forced to counteract the track movement [37]. 

Many studies have focused on the effects of transport stress on different blood traits [38,39]. Zhang et al. [40] reported that transportation of broilers caused decrease in glycogen in breast and thigh muscles. In addition, transport stress is associated with enhanced skeletal muscle energy metabolism, causing mitochondrial superoxide production, acceleration of lipid peroxidation and the induction of cellular damage [41]. In chickens, stress and kinetic activity before slaughter are also involved in pH variations during the early stages of rigor [42], whereas the final pH of meat mainly depends on the glycogen content at the time of slaughter [43]. 

Thus, time spent in transit to the slaughterhouse is a major concern in terms of welfare and meat quality. The effect of transport duration on animal welfare and the resulting meat quality in broilers have been well researched but data on the interaction between genetic strain and transport duration are sparse. It has been reported that the effect of stress could be different in fast- and slow-growing poultry strains [44]. 

Fast-growing strains tend to produce meat with a slower pH decline, higher ultimate pH and, consequently, greater water-holding capacity [45]. On the other hand, Berri et al. (2007) [46] reported that slow-growing strains suffer more from the lag-phase between catching and slaughter due to their high kinetic activity (i.e., wing flapping) during transport and slaughter. Accordingly, when broilers are subjected to stressful conditions, ‘have been well researched could be different from that in standard broilers. 

Our previous results [47] suggest that a 4-h journey to the slaughterhouse, compared to immediate slaughter in a MPPU, negatively affects some animal welfare traits (tonic immobility, creatine kinase, heterophil/lymphocyte ratio, lysozymes, reactive oxygen species, glucose and haptoglobin) in free-range chickens. The slow-growing chickens showed the highest susceptibility to stress, even with a greater antioxidant defence due to their foraging behaviour. Accordingly, a less stressful slaughtering procedure should be developed for all chicken strains with shorter resting times in the farm, transport and animal storage at the slaughterhouse. This is particularly important for PPP in order to sustain the high welfare standard achieved during life and to maintain meat quality.

## 6. Qualitative and Sanitary Implications

The introduction of a MPPU could have an impact on the quality and hygiene/safety traits of the meat based on three main paradigms: reduction of pre-slaughter stress, transport procedures and proper implementation of the slaughter process (i.e., well-managed small-scale facilities, small number of animals of the same flock slaughtered per day). 

Currently, it is understood that that the reduction of pre-slaughtering stress, especially catching, crating and transport, could affect meat traits. The increased level of epinephrine and glucocorticoids in animals exposed to *ante-mortem* stresses can affect *post-mortem* metabolism and, therefore, meat quality [48]. Pre-slaughtering stress, in particular due to transport, may increase muscle glycogenolysis resulting in glycogen decrease in both breast and thigh muscle [40]. Furthermore, acceleration of lipid peroxidation and induction of muscular cellular damage have been reported after stressful transport, associated with enhanced skeletal muscle energy metabolism and mitochondrial superoxide production [41]. 

Despite there is not a general consensus, these stressful events could therefore affect conversion of muscle to meat and the related protein functionality, following a reduced consumer acceptability and processing functionality of the meat caused by the changes in the water holding capacity, colour, tenderness, texture and shelf-life of meat and derived products [49]. Thigh meat have been reported to be affected more than breast meat by this phenomenon [50]. 

**Figure 4:**
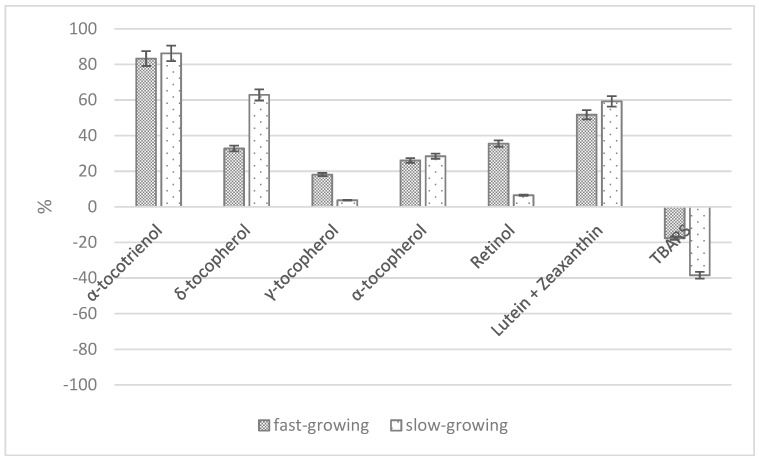
Variation (% with respect to no transport) of antioxidants (α-tocotrienol, α-, γ-, δ-tocopherol, retinol and carotenoids) and TBARS in fast- and slow-growing chicken strains after 4 h of transport (modified by [51]).

As previously reported, studies on the effects of pre-slaughtering practices on meat quality have mainly been conducted in fast-growing broilers, where muscle abnormalities (PSE—Pale, Soft and Exudative and DFD—Dark Firm and Dry condition) were also recorded but when slow-growing strains were considered, they seemed more subjected to stress than fast-growing genotypes due to high kinetic activity during catching, transport and wing-flapping during slaughter [43]. Castellini et al. [51] evaluated the effect of transport duration (0 h vs. 4 h) and chicken genotype (fast- vs. slow-growing strains) reared under free-range conditions. They observed that transport affected the fatty acid profile of breast and drumstick muscle, with a decrease in polyunsaturated fatty acids and antioxidant content (α-tocotrienol, α, δ-tocopherol and carotenoids) and an increase in TBARS (Thiobarbituric acid reactive substances) in breast meat (Figure 4). The decrease in γ-tocopherol, retinol and TBARS was more relevant in birds that were more active, probably due to the higher kinetic activity and the higher peroxidability of their meat. Furthermore, in this study, the breast muscles from 4 h-transported chickens showed significantly less lightness, and also meat tenderness (shear forcevalue) was affected by genotype and transport: meat from slow-growing birds was tougher, whereas after transport, in both genotypes, higher tenderness was observed. Nevertheless, neither PSE nor DFD were recorded.

A PPP system together with a MPPU, when slow-growing strains are used and reduction in the number of chickens to be caught and slaughtered, combined with the absence of transport limits the time spent struggling in crates and, therefore, improves/preserves meat quality.

From a hygiene point of view, there is a large consensus that pre-slaughter stress increases the spread of infectious diseases [37]. The stress that birds experience during pre-slaughter procedures can enhance colonisation by *Campylobacter* spp. [52] and its spread throughout the flock [53].

Previous thinning of the flocks was considered as a major risk factor for contamination of chicken carcasses with *Campylobacter* spp. at the slaughterhouse and catching of the birds for crating further increases *Campylobacter* spp. contamination [54].

In addition, transport vehicles and crates can be considered to be a source of *Campylobacter* contamination [55]. *Campylobacter* from the processing plant can survive on crates for a period sufficient to contaminate the majority of farms in the catchment (it survives for at least 3 h after sanitisation) [56] and poses a contamination risk for uninfected birds belonging to other unrelated flocks [57,58]. Reduction in the time that animals spend in the crates and limiting slaughter to a small number of animals per day that could be caught without prolonged struggling, as well as the absence of transport, could improve the hygiene level of the carcasses.

With regard to *Salmonella* spp., environmental stress could weaken the immune response of birds, with an increase in number of pathogens on the crates [59]. For this reason, reduction in the handling procedures and the absence of transport, as with a MPPU, could strongly influence the prevalence of pathogens at the slaughterhouse. GMP (Good Manufacturing Practices) or guidelines on operator behaviour during pre-slaughter steps could be useful for informing producers about correct handling and crating procedures (with regards to timing, animal density and welfare) to be adopted in MPPU.

Nonetheless, it is reported that the older age of the animals at slaughter, generally adopted in PPP, increased the contamination of caeca by *Campylobacter* spp. [60,61]. Furthermore, when the prevalence of infected animals in the flock is high (i.e., slow-growing genotype with a relatively longer period of rearing), no reduction in *Campylobacter* spp., even without transport, were observed [47]. 

The same consideration was not so for *Salmonella* spp., as different authors reported no shedding animals and no positive carcasses in PPP systems and MPPUs, respectively [47,62]. 

With regard to the slaughter practices in a MPPU, all the procedures are carried out on a manual basis instead of using industrial-scale, automated commercial processing lines [62]. Furthermore, differences in the structures and equipment adopted, as well as in the procedure implemented may strongly affect the hygiene level of the carcasses. For example, in Europe the decontamination strategies could not be used and the limited space available in the truck reduce the possibility of using water-bath chilling with chlorinated water.

Reports on the effect of a MPPU on sanitary traits in poultry meat are scarce [62,63]. It seems likely that the slaughter of a single homogeneous batch of chickens from the same flock during one-day operations could reduce the cross-contamination reported when animals come from different batches and flocks to the same slaughterhouse [54] and, therefore, a daily slaughter rotation of the flocks with a properly cleaned and disinfected MPPU is strongly suggested [64].

Other specific aspects on the possible contamination route inside a MPPU are dependent on structure and equipment. Scalding, defeathering, evisceration and chilling are considered to be the major routes of contamination by both *Salmonella* and *Campylobacter* spp. [65] and have to be carefully considered during HACCP implementation in a MPPU. 

In particular, due to the limited space inside a MPPU, chilling could be carried out in two steps (pre-chilling and chilling) which could be performed within the MPPU and in the farm, respectively [66]. This could have the advantage of allowing the MPPU to be cleaned and disinfected immediately after slaughter, while chilling and storage of carcasses are performed in the farm. 

The use of an air chiller could be more practical for a MPPU, even if counter-flow water-chilling and decontamination strategies, when allowed by national legislation, could be more effective in reducing carcass contamination [62,65]. High carcass density in the chiller could also be avoided to allow proper chilling of the meat and reduce cross-contamination between carcasses [54,67].

In the USA, a technical survey on *Salmonella* and *Campylobacter* showed that *Campylobacter* prevalence was significantly higher in MPPUs and this was partly due to wastewater and compost. In view of this, the processing of waste should be improved for optimum control of human pathogens. 

In Europe, animal by-products must be disposed of as quickly as possible to avoid contamination of the meat for human consumption (Regulation EC 1069/2009), thus providing proper protection of the environment from food-borne pathogens.

The prototype of MPPU shown here was provided by a detailed HACCP manual, with a risk assessment based on hazard probability and severity at each step of the process, validated during the first three months of slaughter and after one year of activity. One of the operators of the MPPU should be responsible for the HACCP plan, including SSOPs. Cleaning and disinfection of the truck and equipment and assessment of the risk of carcass contamination due to scalding, defeathering and evisceration steps (GMPs) and carcass chilling, as the real CCP (Critical Control Point) able to prevent the growth of pathogens, also have to be taken into consideration. 

The absence of *Salmonella* on the carcasses, as well as counts of *Campylobacter* spp., following the criteria lay down by EC Regulation 2073/2005, could be adopted in a MPPU as evidence of the hygiene level, as already performed in conventional industrial slaughterhouses. A reliable carcass sampling could be planned, according to EC Regulation 2073/2005, with 50 samples which should be derived from 10 consecutive sampling sessions

Place and day of slaughtering must be provided to the veterinarian officer to permit Official controls of MPPU. 

## 7. Conclusions

MPPUs are designed to eliminate regulatory impasses and increase marketability and profitability for small-farmers. In addition to economic and technical aspects, there are also other ethical aspects connected with the use of MPPUs.

Nevertheless, positive conclusions exist concerning the effect of MPPUs on animal welfare and product quality. However, inconsistent findings are available regarding sanitary aspects due different equipment and procedures. However, considering that this system practically removes the need to transport poultry and it is used for small quantities of chickens, it is expected that the sanitary aspect can also be improved.

The MPPU could be judged a first step in the development of a new model of alternative poultry production, because it favours different types of change, from farmer to consumers and between the individual stakeholders. In Italy the difficulty to slaughter a low number of animals negatively affect the local productions. The MPPU beyond that to improve the marketing products could increase the connection between the small farms. Developing a network starting from the sharing of the MPPU could ameliorate the collaborations among small farms.

Concerning the Italian poultry sector, the farms have lost her entrepreneurship for the presence of the big companies that control all the production chain. 

According to this view, MPPUs can be considered not only as a solution but also as a resource to emphasise the emergence of food circuits (e.g., farmers’ markets and niche markets) which cannot feature within the global food chain. 

Despite the improvement in poultry welfare (no transport or limited period of transport), the geographical and numerical limitations imposed by EU regulations mean that MPPU development for medium- or large-scale poultry production is unlikely to occur. European legislation has limited further improvement of MPPUs (e.g., different stunning system, carcass decontamination and water-bath chilling), confining PPP meat to local production and selling. Nonetheless, other countries could take advantage of the European experience and improve MPPUs according to national legislation.

In developing countries where the demand for livestock products is strongly increased and in many case the society is organized in small and poorly connected units, the MPPU could represent a real and feasible opportunity of progress.

## Figures and Tables

**Table 1:** Main characteristics of Mobile Poultry Processing Unit vs. conventional slaughterhouse.

Aspects Considered	MPPU		Conventional Slaughtherhouse	
	Pros	Cons	Pros	Cons
**Essential Requirements**	Small truck easily handled	Need of a site for slaughtering in the farm (H_2_o, electricity, etc.)		
		There are numerical and geographic restrictions	No numerical and geographic restrictions	
**Operational and Economic Efficiency**	Sharing the MPPU by farmers allows to reduce the processing cost	Low number of birds processed/hour	High number of birds processed/hour	High unitary cost for transporting live animals (farm–slaughterhouse)
	Possible use of a refrigerated trailer for the delivery of slaughtered carcasses			Cost for transporting carcasses (farm-market)
	Public funds can partially cover MPPU purchase			No public funds for the purchase
**Animal Welfare**	No chicken transport and reduction of pre-slaughter stress			Transport negatively affects animal welfare
**Qualitative and Sanitary Implications**	Safeguarding the quality of meat (low stress)	Good Manufacturing Practices (GMP)for welfare respectful handling and limited time crating		Pre-slaughter stress negatively affects meat quality: change in colour, shelf-life, nutritional parameters
	Low risk of cross contaminations (one flock per day)			Cross contamination due to the processing of different batches of flocks per day Reduction of hygienic/safety condition of meat due to the pre-slaughter stress.
	Reducing carcass contamination due to crating and transport	Difficult in biosafety management (specific for pastured poultry production) Higher age of the birds (Campylobacter)	Proper biosafety measures at flock level	GMP for handling, crating and transport (particularly from different flocks in the same day)
		No decontamination strategy applicable (not allowed in European Union)	Possibility of decontamination strategies of the carcass (water bath chilling)	
